# Clinical Utility of the Triglyceride-Glucose Index in Identifying Poor Glycemic Control in Type 2 Diabetes Mellitus: A Cost-Effective Audit

**DOI:** 10.7759/cureus.109060

**Published:** 2026-05-17

**Authors:** Sajid Ali, Muhammad Muzamil, Umair Ali, Renad Al Mefleh, Roomisa Anis, Muhammad Farooq

**Affiliations:** 1 Cardiology, Punjab Institute of Cardiology, Lahore, PAK; 2 Public Health, Health Services Academy, Islamabad, PAK; 3 Paediatrics, Jordanian Royal Medical Services, Amman, JOR; 4 Biochemistry, Riphah International University, Rawalpindi, PAK; 5 Biochemistry, NUST School of Health Sciences, Islamabad, PAK; 6 Medicine, Watim Medical & Dental College, Rawalpindi, PAK

**Keywords:** glycemic control, insulin resistance, triglycerides, tyg index, type 2 diabetes mellitus

## Abstract

Background: Poor glycemic control in type 2 diabetes mellitus (T2DM) is common and leads to serious complications. The triglyceride-glucose (TyG) index is a simple and low-cost marker that may help identify poor glycemic control using routine tests.

Objective: This study aimed to assess the clinical utility of the TyG index in identifying poor glycemic control in patients with T2DM.

Methods: This observational audit was conducted at the Punjab Institute of Cardiology, Lahore, from February 18, 2025, to February 17, 2026. A total of 412 patients with complete requisite data were analyzed. The TyG index was calculated using fasting triglycerides and fasting blood glucose. Poor glycemic control was defined as HbA1c ≥7.0%. Statistical analysis included group comparison, receiver operating characteristic (ROC) curve analysis, and multivariable logistic regression.

Results: Poor glycemic control was observed in 262 (63.6%) patients. The TyG index was significantly higher in patients with poor glycemic control compared to good glycemic control (9.12 ± 0.54 vs. 8.52 ± 0.41, p < 0.001). ROC analysis showed good diagnostic performance (AUC = 0.81), with an optimal cutoff value of 8.78, sensitivity of 78.2%, and specificity of 72.0%. On multivariable analysis, the TyG index remained an independent predictor of poor glycemic control (AOR = 3.84, 95% CI: 2.45-6.02, p < 0.001). The duration of T2DM and BMI were also significant predictors.

Conclusion: The TyG index is a simple, cost-effective, and reliable marker for identifying poor glycemic control in T2DM patients. It can be useful in routine clinical practice, especially in low-resource settings, where affordability and lack of cutting-edge healthcare facilities are a concern.

## Introduction

Type 2 diabetes mellitus (T2DM) is a major public health problem worldwide, and its burden is rapidly increasing, especially in developing countries like Pakistan [1]. It is a chronic metabolic disease characterized by high blood glucose levels due to insulin resistance and/or reduced insulin secretion. Poor glycemic control in T2DM patients leads to serious complications, such as cardiovascular diseases (CVDs), kidney failure (nephropathy), neuropathy, and retinopathy [2]. These complications increase both morbidity and mortality and place a heavy burden on the healthcare systems [3].

Monitoring glycemic control is very important in the management of diabetes. Glycated hemoglobin (HbA1c) is the most commonly used marker for assessing long-term glycemic control. It reflects the average blood glucose levels over the previous two to three months, or around eight to 12 weeks, as known from the literature [4]. However, HbA1c testing may not always be easily or readily available in low-resource settings. It is relatively expensive and requires standardized or advanced laboratory facilities. In many parts of Pakistan, especially in public hospitals, patients may not undergo regular HbA1c testing due to cost, affordability, or accessibility concerns [5].

Insulin resistance plays a central role in the development and progression of T2DM. It is also associated with abnormal lipid metabolism, particularly elevated triglyceride levels [6]. In recent years, the triglyceride-glucose (TyG) index has gained attention as a simple surrogate marker of insulin resistance. It is calculated using fasting blood glucose and fasting triglyceride levels, both of which are routine and inexpensive laboratory tests. This makes the TyG index a practical tool for clinical use in resource-limited settings [7].

Several international studies have shown that the TyG index is associated with insulin resistance, metabolic syndrome, and cardiovascular risk. Some studies have also suggested a strong relationship between the TyG index and glycemic control in patients with T2DM [8]. A higher TyG index has been linked with higher HbA1c levels and poor metabolic outcomes. However, there is limited local data available from Pakistan to support its clinical use in identifying poor glycemic control [9].

In addition, factors such as duration of diabetes, body mass index (BMI), and demographic characteristics may influence glycemic control [10]. Obesity is a known risk factor for insulin resistance, while longer disease duration is associated with progressive decline in pancreatic beta-cell function. These factors need to be considered when evaluating any surrogate marker [11].

Therefore, this study was designed to assess the clinical utility of the TyG index in identifying poor glycemic control in patients with T2DM in a local population. The aim was to determine whether the TyG index can serve as a reliable and cost-effective alternative to HbA1c in routine clinical practice. This audit may help clinicians in the early identification of high-risk patients and improve diabetes management in resource-limited settings.

## Materials and methods

This observational audit was conducted at the Punjab Institute of Cardiology, Lahore, Pakistan, over a 12-month period from February 18, 2025, to February 17, 2026, and was reported in accordance with STROBE guidelines [12]. Ethical approval was obtained from the institutional Ethical Committee, Punjab Institute of Cardiology, Lahore, Pakistan (ref. no. RTPGME-Research-352, dated February 18, 2025). The study aimed to evaluate the clinical utility of the TyG index as a cost-effective surrogate marker for identifying poor glycemic control in patients with T2DM. A cross-sectional analytical design was adopted, enrolling adult patients presenting to outpatient and inpatient departments.

The sample size was calculated using a single population proportion formula, assuming a prevalence of poor glycemic control of 50% to ensure maximum variability, a 95% confidence level, and a 5% margin of error, yielding a minimum required sample of 384 participants; after accounting for an anticipated 10% incomplete data rate, a total of 425 patients were targeted. Consecutive non-probability sampling was used to recruit eligible participants until the required sample size was achieved. Inclusion criteria comprised patients aged 30-75 years with a confirmed diagnosis of T2DM for at least six months and availability of FBG, fasting triglycerides (TG), and glycated hemoglobin (HbA1c) values within the same visit. Patients with type 1 diabetes, gestational diabetes, acute illness, chronic liver disease, end-stage renal disease, thyroid dysfunction, use of lipid-altering drugs initiated within the past three months, or incomplete laboratory records were excluded to minimize bias.

Data were extracted from hospital records using a standardized proforma. Demographic variables included age, gender, duration of diabetes, BMI, and treatment modality. Laboratory parameters included FBG (mg/dL), fasting TG (mg/dL), and HbA1c (%), measured using routine hospital assays following standard operating procedures. The TyG index was calculated using the formula as follows: fasting TG (mg/dL) × fasting glucose (mg/dL) / 2. Poor glycemic control was operationally defined as HbA1c ≥7.0% in accordance with international diabetes guidelines. Potential confounders such as age, BMI, duration of diabetes, and use of antidiabetic medications were recorded and adjusted for in the analysis.

Data quality was ensured through double-entry verification and random cross-checking of 10% of records. Missing data were handled using complete case analysis; records with missing key variables (FBG, TG, or HbA1c) were excluded, while minor missing demographic variables were imputed using mean or median values where appropriate. Statistical analysis was performed using IBM SPSS Statistics for Windows, Version 26.0 (released 2019, IBM Corp., Armonk, NY). Continuous variables were tested for normality using the Shapiro-Wilk test and expressed as mean ± standard deviation or median (interquartile range) as appropriate, while categorical variables were presented as frequencies and percentages. Independent sample t-test (Parametric) or Mann-Whitney U test (non-parametric) was used to compare TyG index between groups with good and poor glycemic control. Receiver operating characteristic (ROC) curve analysis was performed to determine the diagnostic performance of the TyG index and to identify the optimal cutoff value using the Youden index. Multivariable logistic regression analysis was conducted to assess the independent association of TyG index with poor glycemic control after adjusting for confounders, with results reported as adjusted odds ratios (AORs) and 95% confidence intervals. A p-value of <0.05 was considered statistically significant.

## Results

A total of 425 patients with T2DM were enrolled, of whom 412 had complete datasets and were included in the final analysis after exclusion of 13 records due to missing key laboratory values. The mean age of the participants was 54.2 ± 9.8 years, with a slight male predominance (56.3%). Poor glycemic control (HbA1c ≥7.0%) was observed in 262 (63.6%) patients, while 150 (36.4%) had adequate control.

Table 1 summarizes the baseline demographic and clinical characteristics of the study population stratified by glycemic control status. Patients with poor glycemic control had a significantly longer duration of diabetes, higher BMI, and higher fasting blood glucose levels compared to those with good control (p < 0.05).

**Table 1:** Baseline characteristics of the study participants according to the glycemic control status (n = 412)

Variable	Total (n = 412)	Good control (n = 150)	Poor control (n = 262)	p-value	Reference range / normal value
Age (years, mean ± SD)	54.2 ± 9.8	53.6 ± 9.5	54.6 ± 10.0	0.312	No clinical reference range
Male, n (%)	232 (56.3)	80 (53.3)	152 (58.0)	0.358	Not applicable
Duration of diabetes (years, mean ± SD)	8.6 ± 4.7	7.2 ± 4.1	9.4 ± 4.9	<0.001	No clinical reference range
BMI (kg/m², mean ± SD)	27.8 ± 4.3	26.9 ± 3.9	28.4 ± 4.4	0.002	Normal: 18.5–24.9 kg/m²; overweight: 25.0–29.9; obese: ≥30
Fasting blood glucose (mg/dL, mean ± SD)	162.5 ± 48.2	121.3 ± 22.6	186.7 ± 45.9	<0.001	Normal: 70–99 mg/dL; prediabetes: 100–125; diabetes: ≥126
Triglycerides (mg/dL, median (IQR))	178 (140–225)	152 (128–190)	195 (155–245)	<0.001	Normal: <150 mg/dL; borderline high: 150–199; high: 200–499
HbA1c (% , mean ± SD)	7.9 ± 1.4	6.4 ± 0.4	8.7 ± 1.2	<0.001	Normal: <5.7%; prediabetes: 5.7–6.4%; diabetes: ≥6.5%; good diabetic control: <7.0%

The TyG index was significantly higher in patients with poor glycemic control compared to those with good glycemic control (9.12 ± 0.54 vs. 8.52 ± 0.41, p < 0.001), as shown in Table 2.

**Table 2:** Comparison of the TyG index between the glycemic control groups

Variable	Good control (n = 150)	Poor control (n = 262)	p-value
TyG index (mean ± SD)	8.52 ± 0.41	9.12 ± 0.54	<0.001

ROC curve analysis demonstrated that the TyG index had good discriminative ability in identifying poor glycemic control, with an area under the curve (AUC) of 0.81 (95% CI: 0.76-0.85, p < 0.001). The optimal cutoff value of the TyG index was 8.78, yielding a sensitivity of 78.2% and specificity of 72.0%, as shown in Table 3.

**Table 3:** ROC analysis of TyG index for predicting poor glycemic control ROC: receiver operating characteristic, TyG: triglyceride-glucose, AUC: area under the curve

Parameter	Value
AUC (95% CI)	0.81 (0.76–0.85)
Optimal cutoff (TyG index)	8.78
Sensitivity (%)	78.2
Specificity (%)	72.0
Positive predictive value (%)	83.5
Negative predictive value (%)	64.8
p-value	<0.001

Multivariable logistic regression analysis revealed that the TyG index remained an independent predictor of poor glycemic control after adjusting for age, BMI, duration of diabetes, and treatment modality. Each unit increase in the TyG index was associated with a significantly higher likelihood of poor glycemic control (AOR: 3.84, 95% CI: 2.45-6.02, p < 0.001). Duration of diabetes and BMI were also independently associated with poor glycemic control (p < 0.05), as presented in Table 4.

**Table 4:** Multivariable logistic regression analysis for the predictors of poor glycemic control

Variable	AOR	95% CI	p-value
TyG index	3.84	2.45-6.02	<0.001
Age (years)	1.01	0.98-1.03	0.421
BMI (kg/m²)	1.08	1.02-1.15	0.009
Duration of diabetes (years)	1.12	1.06-1.18	<0.001
Male gender	1.19	0.78-1.81	0.417

## Discussion

This study highlights the importance of using simple and low-cost markers for assessing glycemic control in T2DM. In many healthcare settings across Pakistan, tests such as HbA1c may not always be easily available or affordable. Therefore, evaluating the TyG index as an alternative is clinically useful and relevant.

In the present study, most patients had poor glycemic control, which is similar to previous reports where a large proportion of diabetic patients fail to achieve target HbA1c levels. This reflects poor disease control and possible issues like delayed diagnosis, poor compliance, and limited access to healthcare. Studies have also shown that poor glycemic control is very common in developing countries due to the aforementioned factors [13].

Regarding demographic and clinical parameters, we found that duration of diabetes and BMI were significantly higher in patients with poor glycemic control. This is supported by published literature, where a longer duration of diabetes is associated with progressive beta-cell dysfunction and worsening or deteriorating glycemic control [14]. Similarly, higher BMI reflects obesity, which is a known cause of insulin resistance and poor glucose regulation [15]. Previous studies have also shown that BMI and duration of diabetes are significantly higher in patients with uncontrolled diabetes [16].

Fasting blood glucose and triglyceride levels were also significantly elevated in patients with poor glycemic control. This finding is expected because fasting glucose directly reflects short-term glycemic status, while elevated triglycerides indicate underlying metabolic dysfunction and insulin resistance [17]. Studies have shown that both fasting glucose and triglycerides are significantly higher in patients with poor glycemic control, and both contribute to the calculation of the TyG index [18].

The most important finding of our study was that the TyG index was significantly higher in patients with poor glycemic control. This is consistent with multiple studies showing that the TyG index is strongly associated with HbA1c and insulin resistance [19]. The TyG index combines fasting glucose and triglycerides, so it reflects both glycemic status and lipid metabolism. Previous research has shown a strong positive correlation between TyG index and HbA1c levels, indicating that higher TyG values are associated with worsening glycemic control [20]. The logical explanation is that insulin resistance leads to both increased glucose production and impaired lipid metabolism, resulting in elevated triglycerides and glucose levels simultaneously [18].

Our ROC analysis showed good diagnostic performance of the TyG index, with an AUC of 0.81. This is comparable to earlier studies where AUC values around 0.74 to 0.88 have been reported, indicating good predictive ability of the TyG index for poor glycemic control. The sensitivity and specificity values in our study also support its usefulness as a screening tool. This means that the TyG index can reasonably identify patients with poor glycemic control without requiring expensive tests [18] [17].

In multivariable analysis, the TyG index remained an independent predictor of poor glycemic control even after adjusting for confounders. This strengthens the evidence that TyG is not just associated but independently related to poor glycemic status. Similar findings have been reported in previous studies where the TyG index showed a strong independent association with glycemic control and insulin resistance [16]. Duration of diabetes and BMI also remained significant, which again supports their role in disease progression and metabolic imbalance [21].

From a clinical perspective, the TyG index is very useful because it uses routine laboratory tests that are cheaper and readily and widely available. It can help clinicians identify high-risk patients early, especially in resource-limited settings. However, this study has some limitations. It is a single-center study and uses a cross-sectional design, so causal relationships cannot be established. Some confounders, like diet and physical activity, were not assessed. Future studies should include multicenter data and longitudinal follow-up to validate cutoff values and clinical applicability.

## Conclusions

The TyG index is a simple, cost-effective, and reliable marker for identifying poor glycemic control in patients with T2DM. It shows good diagnostic accuracy and a strong association with HbA1c and metabolic parameters. It can be used as a practical alternative tool in routine clinical practice, especially in low-resource settings.
